# Behavioral effects of *Bj*-PRO-7a, a proline-rich oligopeptide from *Bothrops jararaca* venom

**DOI:** 10.1590/1414-431X20198441

**Published:** 2019-11-07

**Authors:** L.C. Turones, K.R. da Cruz, G. Camargo-Silva, L.L. Reis-Silva, D. Graziani, P.M. Ferreira, P.M. Galdino, G.R. Pedrino, R. Santos, E.A. Costa, D. Ianzer, C.H. Xavier

**Affiliations:** 1 Laboratório de Neurobiologia de Sistemas, Departamento de Ciências Fisiológicas, Instituto de Ciências Biológicas, Universidade Federal de Goiás, Goiânia, GO, Brasil; 2 Laboratório de Farmacologia de Produtos Naturais e Sintéticos, Departamento de Farmacologia, Instituto de Ciências Biológicas, Universidade Federal de Goiás, Goiânia, GO, Brasil; 3 Departamento de Fisiologia e Biofísica, Universidade Federal de Minas Gerais, Belo Horizonte, MG, Brasil

**Keywords:** *Bj*-PRO-7a, Snake venom, Neuroactive compounds, Anxiety, Depression, Behavior

## Abstract

The heptapeptide *Bj*-PRO-7a, isolated and identified from *Bothrops jararaca* (*Bj*) venom, produces antihypertensive and other cardiovascular effects that are independent on angiotensin converting enzyme inhibition, possibly relying on cholinergic muscarinic receptors subtype 1 (M_1_R). However, whether *Bj*-PRO-7a acts upon the central nervous system and modifies behavior is yet to be determined. Therefore, the aims of this study were: i) to assess the effects of acute administration of *Bj*-PRO-7a upon behavior; ii) to reveal mechanisms involved in the effects of *Bj*-PRO-7a upon locomotion/exploration, anxiety, and depression-like behaviors. For this purpose, adult male Wistar (WT, wild type) and spontaneous hypertensive rats (SHR) received intraperitoneal injections of vehicle (0.9% NaCl), diazepam (2 mg/kg), imipramine (15 mg/kg), *Bj*-PRO-7a (71, 213 or 426 nmol/kg), pirenzepine (852 nmol/kg), α-methyl-DL-tyrosine (200 mg/kg), or chlorpromazine (2 mg/kg), and underwent elevated plus maze, open field, and forced swimming tests. The heptapeptide promoted anxiolytic and antidepressant-like effects and increased locomotion/exploration. These effects of *Bj*-PRO-7a seem to be dependent on M_1_R activation and dopaminergic receptors and rely on catecholaminergic pathways.

## Introduction

Anxiety and depression are considered the principal debilitating diseases in the world, capable of compromising human wellness. Both diseases are highly prevalent and, as a consequence, there are substantially negative social and economic impacts. Because of the global incidence, developing new and efficient drugs is worth the effort, as the therapeutic arsenal currently available either displays delayed effects or shows no response in many cases of anxiety and depression (1). Therefore, venoms may represent a rich source of promising molecules, which includes neuroactive compounds and polypeptides (2).

Seminal research with the venom of *Bothrops jararaca* (*Bj*), a pit viper broadly distributed in South America, resulted in breakthrough discoveries of two compounds: bradykinin potentiating factors (BPFs) and bradykinin (Bk) itself. Subsequently, these BPFs were isolated and identified as peptides, formerly named bradykinin potentiating peptides (BPPs). Nevertheless, some BPPs were not capable of potentiating the Bk effects in pharmacological studies and were thus renamed as proline-rich oligopeptides (PROs) according to the similarity in their primary structure. The *Bj*-PROs have 5–17 amino acids residues with proline residues at C-terminal and pyroglutamic acid at N-terminal. The latter is a feature that confers high resistance against proteolytic activity exerted by some enzymes [for review, see Camargo et al. (3)].

As reported in the review by Camargo et al. (3), the first peptides identified by the scientists Sergio Henrique Ferreira and colleagues (1970) were capable of inhibiting the catalytic site of angiotensin converting enzyme (ACE), as shown by *in vitro* assays. Following the advances that uncovered the importance of the renin-angiotensin system to the cardiovascular homeostasis, several *Bj*-PROs analogues were synthesized and tested, presenting therapeutic potential to treat hypertension and other cardiovascular diseases. One of these molecules, captopril, presented major oral activity and was the first ACE inhibitor (ACEi) commercially available. However, this ACEi presented side effects, which encouraged further research. The study of the two ACE domains by Wei and coworkers and the discovery of *Bj*-PROs new sequences allowed for additional progress (3). Newly described peptides such as *Bj*-PROs 7a, 11e, and 12b do not present affinity to ACE C-terminal domain, which is responsible for converting angiotensin I to angiotensin II. Intriguingly, these peptides evoked antihypertensive effects that were concomitant to bradycardia, through mechanisms that go far beyond those involving ACE inhibition (4).

*Bj*-PRO-7a, identified and isolated in the *Bj* crude venom, is composed by seven amino acids residues with an aspartic residue in N-terminal position (Supplementary Figure S1) (5). The heptapeptide presented a potent and long-lasting anti-hypertensive effect in spontaneous hypertensive rats (SHR) that did not involve ACE (6). In addition, the heptapeptide was found intact in mice urine 24 h after intraperitoneal (*ip*) injection, which suggests a high resistance to enzymatic degradation (7). Another study demonstrated that *Bj*-PRO-7a modulates calcium transients in neurons and activates cholinergic muscarinic receptors subtype 1 (M_1_R) *in vitro* (8). Therefore, *Bj*-PRO-7a is able to act upon multiple targets and possibly affect centrally organized functions. In this regard, the hypothesis we raised is that *Bj*-PRO-7a would modify the behavior also by cholinergic mechanisms. Additionally, we tested whether *Bj*-PRO-7a effects would rely on catecholaminergic paths, since catecholamines also play a role in the etiology of depression and anxiety and are frequently chosen as targets for pharmacological treatments (9). To assess whether the possible central effects evoked by the heptapeptide would be independent of cardiovascular effects, hypertensive and normotensive rats were used. The aim of this study was to assess *Bj*-PRO-7a effects on locomotion, exploration, anxiety, and depression-like behaviors, and the possible mechanisms involved.

## Material and Methods

### General procedures

#### Animals

Male normotensive Wistar rats (WT) and SHR (weighing 300–380 g) bred at the Central Animal Facility of the Federal University of Goiás (UFG, Brazil) were randomly allocated to polypropylene cages (47×31×16 cm), five rats per cage, with water and food *ad libitum*, under controlled temperature and light/dark cycle of 12/12 h. Systolic blood pressure (SBP) was measured by tail plethysmography in SHRs to confirm hypertension (SHR: 194±7 *vs* WT: 136±6 mmHg; P<0.05). When the animals reached 15 weeks of age, SBP was recorded for 4 days. The rats were pre-warmed to approximately 38°C for 15 min to dilate the tail artery. An inflatable cuff and pressure receptor were placed on the tail; both were coupled to a transducer and connected to an acquisition data system (PowerLab 4/25, ML0380/D, ADInstruments, Australia). The arterial pulse was measured by the inflatable cuff.

All experiments were approved by our local institutional animal welfare committee (CEUA/UFG protocol number 018/2015) and were performed in accordance with the Brazilian Federal Law No. 11.794. We made all efforts to minimize the number of animals used.

#### Drugs and reagents

All drugs were diluted in sterile saline (0.9% NaCl), which was the vehicle of choice. The drugs tested were: i) *Bj*-PRO-7a (GenOne Biotechnology, Brazil) at doses of 71, 213, or 426 nmol/kg already tested in a previous study (6); ii) the specific antagonist of M_1_R pirenzepine (Sigma, USA) at the dose of 852 nmol/kg; iii) the benzodiazepine diazepam (Sigma) at the dose of 2 mg/kg, used as positive control for anxiety and locomotion tests; iv) the tricyclic antidepressant imipramine (Sigma) at the dose of 15 mg/kg, used as positive control in depressive tests; v) the inhibitor of tyrosine hydroxylase α-methyl-DL-tyrosine (AMPT) (Sigma) at the dose of 200 mg/kg, used to reveal the catecholamines contribution; vi) the non-specific dopaminergic antagonist chlorpromazine (Sigma) at the dose of 2 mg/kg, used to assess the possible contribution of dopamine receptors. All drugs and vehicle were injected *ip.*

### Behavioral tests

On the experimental day, the rats were transported from the sectorial animal facility to the laboratory where experiments were performed and allocated in a separate room during 1 h for adaptation. Then, rats received vehicle or drugs according to the experiments – these administrations were conducted by the same person to avoid handling stress. All experiments were performed in a separate, quiet, and dimly illuminated room to prevent animals' communication and possible interferences during the experimental trials.

#### Elevated plus maze (EPM)

Anxiety was evaluated with the EPM test (10). The maze consisted of two open arms (50×10×1 cm) and two closed arms (50×10×40 cm) connected by a central platform (10×10 cm). The maze was elevated 50 cm from the ground and a video camera was fixed above the maze to record the rats' movements for posterior analysis. The maze was cleaned with 10% alcohol between experiments to prevent olfactory clues. At the start of the trial, each rat was placed in the center of the maze facing one of the closed arms. Animals were allowed to explore the maze for 5 min. Six variables were measured: 1) time spent in the open arms; 2) number of entries into the open arms; 3) time spent in the closed arms (the placement of all four paws in the arms); 4) number of entries into the closed arms; 5) time spent in the central platform; and 6) number of total entries. A higher time and frequency in open arms, compared to vehicle, indicated an anxiolytic effect. In addition, the number of entries into the closed arms and the total entries also indicated general activity or locomotor activity, which was confirmed in the open field test.

#### Open field test (OF)

Locomotion and exploration were evaluated in the OF test (10). The field was a round arena (120 cm in diameter) surrounded by a wall of 40 cm, with the surface subdivided into 12 squares. A video camera was fixed above the field to record the rats' movements for posterior analysis. The OF test was performed immediately after the EPM test. The field was cleaned with 10% alcohol between experiments to prevent olfactory clues. Each rat was placed in the center of the field and was allowed to explore the apparatus for 5 min. Six variables were measured: 1) time spent in the periphery; 2) time spent in the center; 3) immobility time; 4) number of square crossings; 5) number of rearing episodes; and 6) number of self-grooming (including paw licking, nose/face grooming, and head washing). Immobility, crossings, and self-grooming were the locomotor outcomes; rearing episodes were recorded as exploratory behavior.

#### Forced swimming (FS)

The FS test was performed as previously described (10). On the first day, rats were placed in a cylinder of polyvinyl chloride (24 cm of diameter and 60 cm of high) filled by a water column (42 cm at 25±1°C) and were allowed to swim for 15 min. Then, the rats were dried and placed in a warmed cage at approximately 38°C. Subsequently, the first drug administration (24 h before FS test) was performed. On the next day, the second and third drug administrations were performed, 5 and 1 h before the FS test, respectively. During the test, each rat was allowed to swim for 6 min. The experiment was recorded by a video camera fixed above the cylinder and the last 4 min were used for posterior analysis. The cylinder was cleaned with 10% alcohol and the water was removed after each experiment to prevent olfactory clues. The immobility time (when the rat stopped the struggling movements and floated on the water, making only movements to keep the head above the water surface) was a measure of depressive behavior.

### Experimental design

#### Experiment 1: Effects of Bj-PRO-7a on anxiety and locomotion/exploration

WT and SHR received an *ip* injection of vehicle, diazepam, or *Bj*-PRO-7a (71, 213, and 426 nmol/kg) 30 min before the EPM test. After the end of EPM testing, rats were tested in the OF.

#### Experiment 2: Involvement of central M_1_R in the responses evoked by Bj-PRO-7a on anxiety and locomotion/exploration

We used a systemic (*ip*) injection of pirenzepine to maintain the same route of administration for all drugs, which also ensured a minimally invasive method. Although pirenzepine presents low liposolubility, previous studies demonstrated that it crosses the blood brain barrier, reaches the central nervous system (11), and produces central effects (12,13).

WT rats received pirenzepine (852 nmol/kg, *ip*) 30 min before injection of vehicle or *Bj*-PRO-7a (426 nmol/kg, *ip*). Thirty min after the last injection (vehicle or *Bj*-PRO-7a, *ip*), the EPM test was performed. Following the end of EPM, rats were tested in the OF.

#### Experiment 3: Involvement of catecholaminergic pathways in the effects evoked by Bj-PRO-7a on anxiety and exploration/locomotion

WT rats received AMPT (200 mg/kg, *ip*) 1 h before an injection of vehicle or *Bj*-PRO-7a (426nmol/kg, *ip*). Thirty min after vehicle or *Bj*-PRO-7a administration the EPM test was performed. Following the end of EPM, rats were tested in the OF.

#### Experiment 4: Contribution of dopaminergic receptors to the effects evoked by Bj-PRO-7a on anxiety and exploration/locomotion

WT rats received chlorpromazine (2 mg/kg, *ip*) 30 min before vehicle or *Bj*-PRO-7a (426 nmol/kg, *ip*). Thirty min after vehicle or *Bj*-PRO-7a administration the EPM test was performed followed by the OF.

#### Experiment 5: Effects of Bj-PRO-7a on depression-like behavior

WT rats and SHRs received three injections of vehicle, imipramine (15 mg/kg, *ip*), or *Bj*-PRO-7a (71, 213 or 426 nmol/kg, *ip*) 24, 5, and 1 h before the FS test.

#### Experiment 6: Involvement of M_1_R in the responses evoked by Bj-PRO-7a on depression-like behavior

WT rats received three injections of vehicle or *Bj*-PRO-7a (426 nmol/kg, *ip*) 24, 5, and 1 h before the FS test. Pirenzepine (852 nmol/kg, *ip*) was given 30 min before the last vehicle or *Bj*-PRO-7a administration.

#### Experiment 7: Involvement of catecholaminergic pathways in the responses evoked by Bj-PRO-7a on depression-like behavior

WT rats received three injections of vehicle or *Bj*-PRO-7a (426 nmol/kg, *ip*) at 24, 5, and 1 h before the FS test. α-methyl-DL-tyrosine (200 mg/kg, *ip*) was injected 1 h before the last vehicle or *Bj*-PRO-7a administration.

#### Experiment 8: Contribution of dopaminergic receptors to the responses evoked by Bj-PRO-7a on depression-like behavior

WT rats received three injections of vehicle or *Bj*-PRO-7a (426 nmol/kg, *ip*) at 24, 5, and 1 h before the FS test. Chlorpromazine (2 mg/kg, *ip*) was injected 30 min before the last vehicle or *Bj*-PRO-7a administration.

### Statistical analysis

The data are reported as means±SE and were analyzed by two-way ANOVA followed by Bonferroni's *post hoc* test (experiments 1 and 5, factors strain and treatment) or one-way ANOVA followed by Fisher's *post hoc* test (experiments 2–4 and 6–8). Standard deviation was set as the exclusion parameter for statistical analysis. All data were analyzed using GraphPad Prism 6.0 (GraphPad Software, Inc., USA). The level of significance was fixed at P<0.05.

## Results

### *Bj-*PRO-7a produced an anxiolytic-like effect and locomotor/exploratory responses

**Figure 1.**
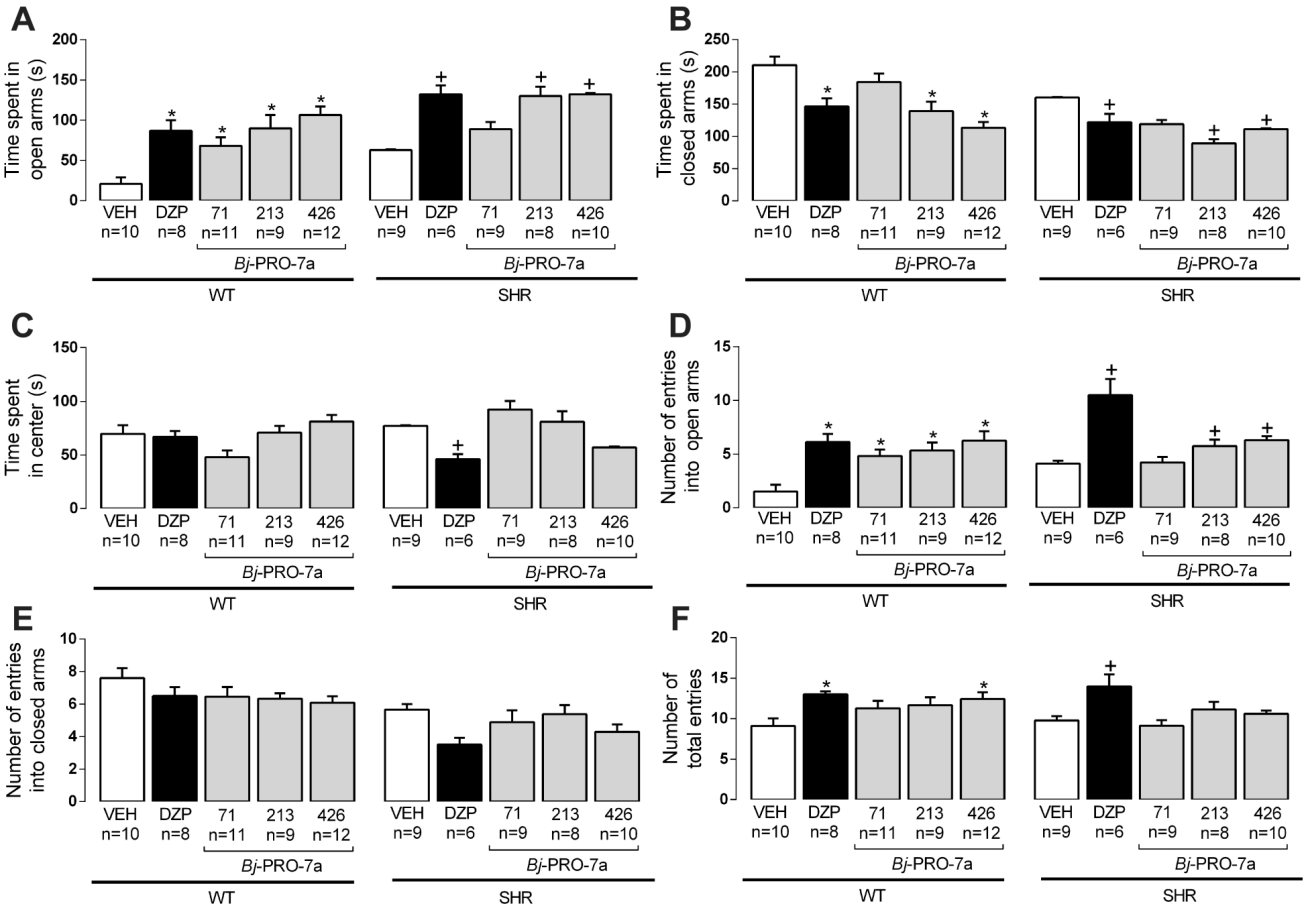
Effects of Bj-PRO-7a on anxiety-like behaviors in the elevated plus maze in Wistar (WT) and spontaneously hypertensive rats (SHRs). **A**, Time spent in open arms; **B**, Time spent in closed arms; **C**, Time spent in center; **D**, Number of entries into open arms; **E**, Number of entries into closed arms; **F**, Number of total entries. Experimental groups: VEH: vehicle (0.9% NaCl); DZP: diazepam (2 mg/kg); Bj-PRO-7a (71, 213, or 426 nmol/kg). Data are reported as means±SE. *P<0.05 *vs* VEH WT; ^+^P<0.05 *vs* VEH SHR (two-way analysis of variance followed by Bonferroni's *post hoc* test).

As expected, the treatment with diazepam increased the time and the number of entries in open arms in WT rats (Figure 1A and D). The same anxiolytic effect was observed in SHRs. *Bj*-PRO-7a decreased anxiety in WT rats, as revealed by the increases in the time and number of entries into open arms of the maze (Figure 1A and D). Similar effects were observed in the time spent in open arms by SHRs injected with the heptapeptide and in the number of entries into open arms, except at the dose of 71 nmol/kg. *Bj*-PRO-7a also reduced the time spent in closed arms, except for the dose of 71 nmol/kg (Figure 1B). The treatment with *Bj*-PRO-7a did not change the number of entries into the closed arms (Figure 1E). Interestingly, the total number of entries was increased by *Bj*-PRO-7a at doses of 426 nmol/kg in WT rats (Figure 1F). The time spent in the maze center did not present significant changes with *Bj*-PRO-7a treatment.

**Figure 2.**
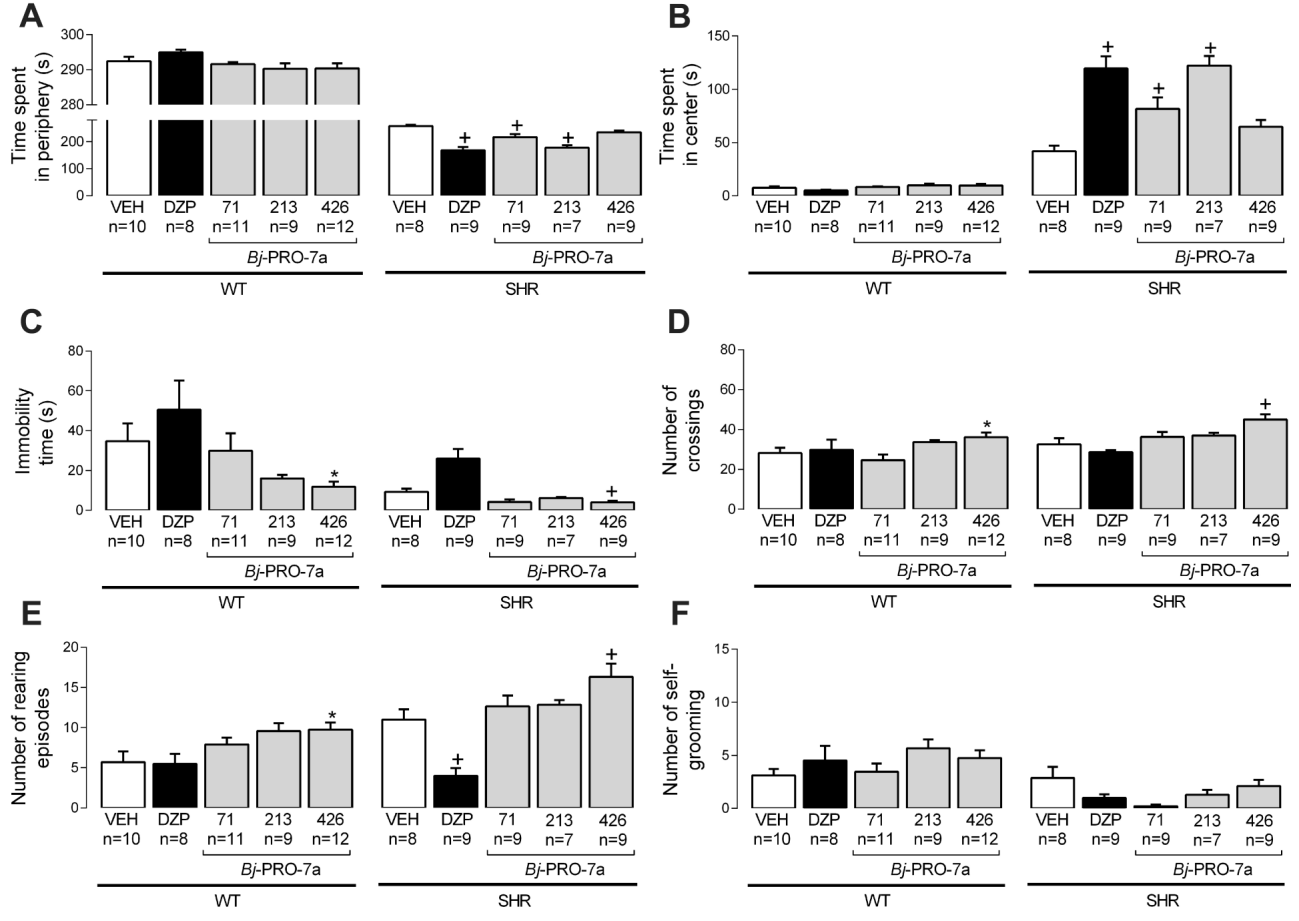
Effects of Bj-PRO-7a on locomotion and exploration activities in the open field in Wistar (WT) and spontaneously hypertensive rats (SHRs). **A**, Time spent in periphery; **B**, Time spent in center; **C**, Immobility time; **D**, Number of crossings; **E**, Number of rearing episodes; **F**, Number of self-grooming episodes. Experimental groups: VEH: vehicle (0.9% NaCl); DZP: diazepam (2 mg/kg); Bj-PRO-7a (71, 213, or 426 nmol/kg). Data are reported as means±SE. *P<0.05 *vs* VEH WT; ^+^P<0.05 *vs* VEH SHR (two-way analysis of variance followed by Bonferroni's *post hoc* test).

The treatment with *Bj*-PRO-7a reduced the time spent in the periphery (Figure 2A) and increased the time spent in the center (Figure 2B) in SHRs, but not in WT rats. Additionally, the treatment with *Bj*-PRO-7a at 426 nmol/kg increased locomotion and exploration, as revealed by reductions in the immobility time (Figure 2C), increases in the number of crossings (Figure 2D), and rearing episodes (Figure 2E). The heptapeptide did not significantly modify the self-grooming (Figure 2F).

Since the results obtained from these first experiments showed small differences in the effects of *Bj*-PRO-7a between strains, the subsequent trials were conducted only in WT rats. Considering that 426 nmol/kg *Bj*-PRO-7a was the dose that produced pronounced effects, this dose was chosen to investigate the subsequent mechanistic hypothesis using pirenzepine (PZP), α-methyl-DL-tyrosine (AMPT), and chlorpromazine (CLP).

### Activation of muscarinic receptor subtype 1 was important to the anxiolysis promoted by *Bj*-PRO-7a

**Figure 3.**
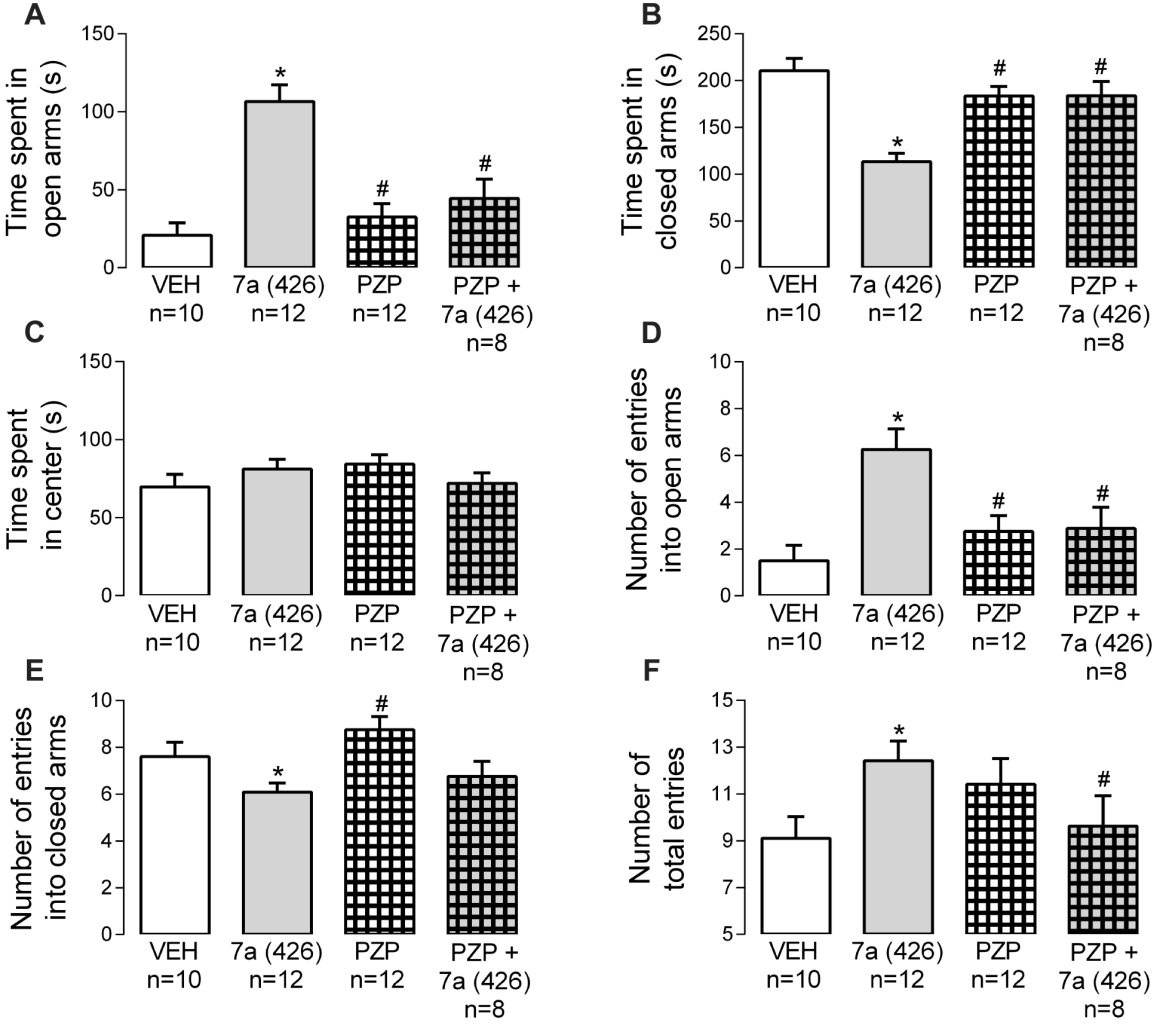
Contribution of muscarinic receptor subtype 1 (as revealed by the antagonism with pirenzepine) to the anxiety-like effect evoked by Bj-PRO-7a in the elevated plus maze. **A**, Time spent in open arms; **B**, Time spent in closed arms; **C**, Time spent in center; **D**, Number of entries into open arms; **E**, Number of entries into closed arms; **F**, Number of total entries. Experimental groups: VEH: vehicle (0.9% NaCl); 7a (426): Bj-PRO-7a (426 nmol/kg); PZP: pirenzepine (852 nmol/kg); PZP+7a (426): co-administration of pirenzepine and Bj-PRO-7a (426 nmol/kg). Data are reported as means±SE. *P<0.05 *vs* vehicle; ^#^P<0.05 *vs* 7a (426) (one-way analysis of variance followed by Fisher's *post hoc* test).

**Figure 4.**
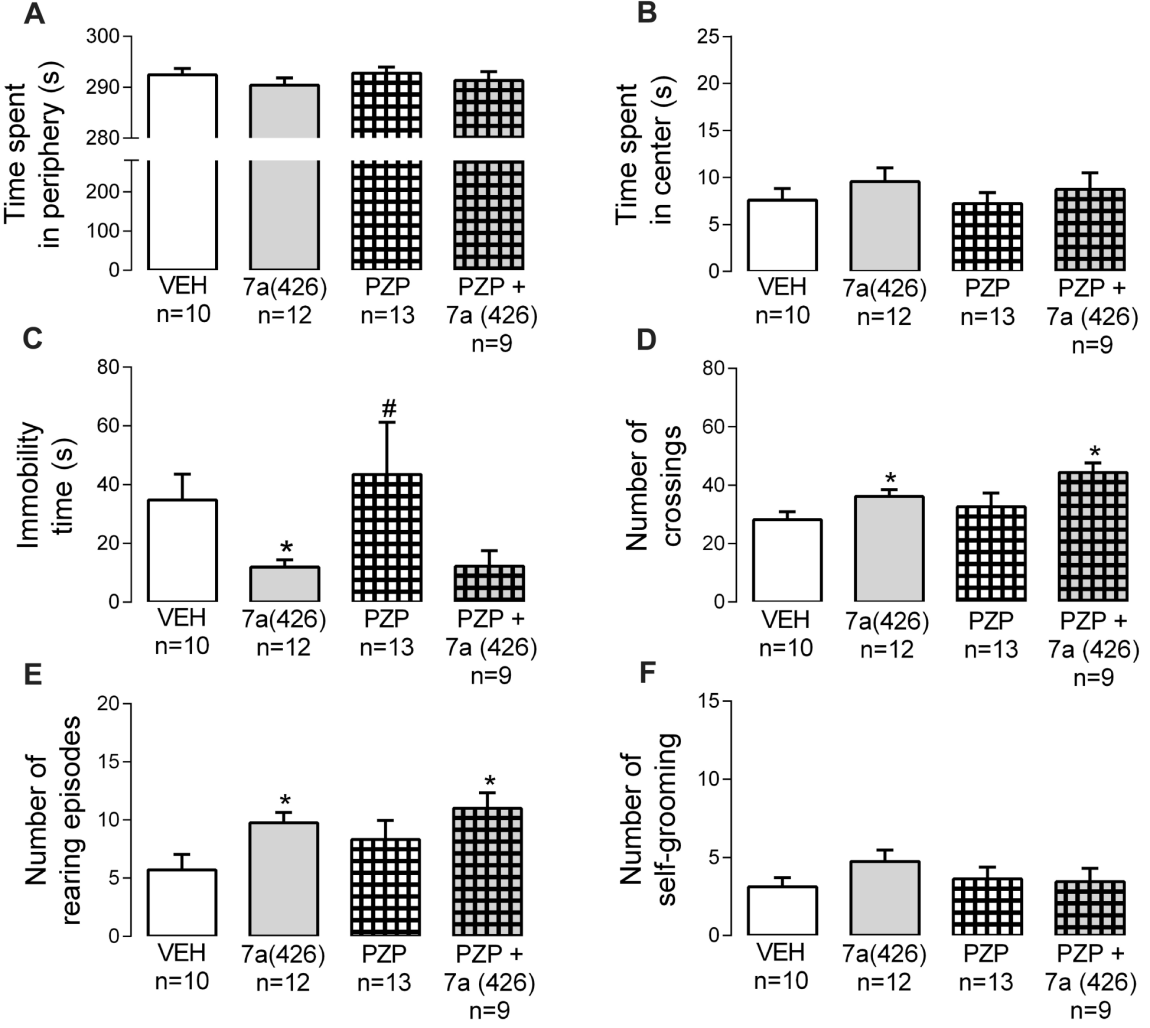
Contribution of muscarinic receptor subtype 1 (as revealed by the antagonism with pirenzepine) to the effects evoked Bj-PRO-7a on locomotion/exploration in the open field. **A**, Time spent in periphery; **B**, Time spent in center; **C**, Immobility time; **D**, Number of crossings; **E**, Number of rearing episodes; **F**, Number of self-grooming episodes. Experimental groups: VEH: vehicle (0.9% NaCl); 7a (426): Bj-PRO-7a (426 nmol/kg); PZP: pirenzepine (852 nmol/kg); PZP + 7a (426): co-administration of PZP and Bj-PRO-7a (426 nmol/kg). Data are reported as means±SE. *P<0.05 *vs* vehicle; ^#^P<0.05 *vs* 7a (426) (one-way analysis of variance followed by Fisher's *post hoc* test).

PZP (852 nmol/kg) did not promote significant alterations in EPM and OF tests compared to the vehicle group (Figures 3 and 4). Interestingly, the combined injection of *Bj*-PRO-7a with PZP reverted the increases in the time and number of entries into the open arms of EPM evoked by *Bj*-PRO-7a (Figure 3A and D). In addition, PZP reverted the reduction in the time spent in closed arms produced by the heptapeptide (Figure 3B). This demonstrated that the anxiolytic-like effect of *Bj*-PRO-7a depends on M_1_R. However, the effects of *Bj*-PRO-7a (reduction of immobility time, increase in crossing and rearing episodes) in the OF test were not modified by the antagonism of M_1_R (Figure 4C-F).

### The catecholaminergic system was involved in anxiolytic-like effects evoked by *Bj*-PRO-7a

**Figure 5.**
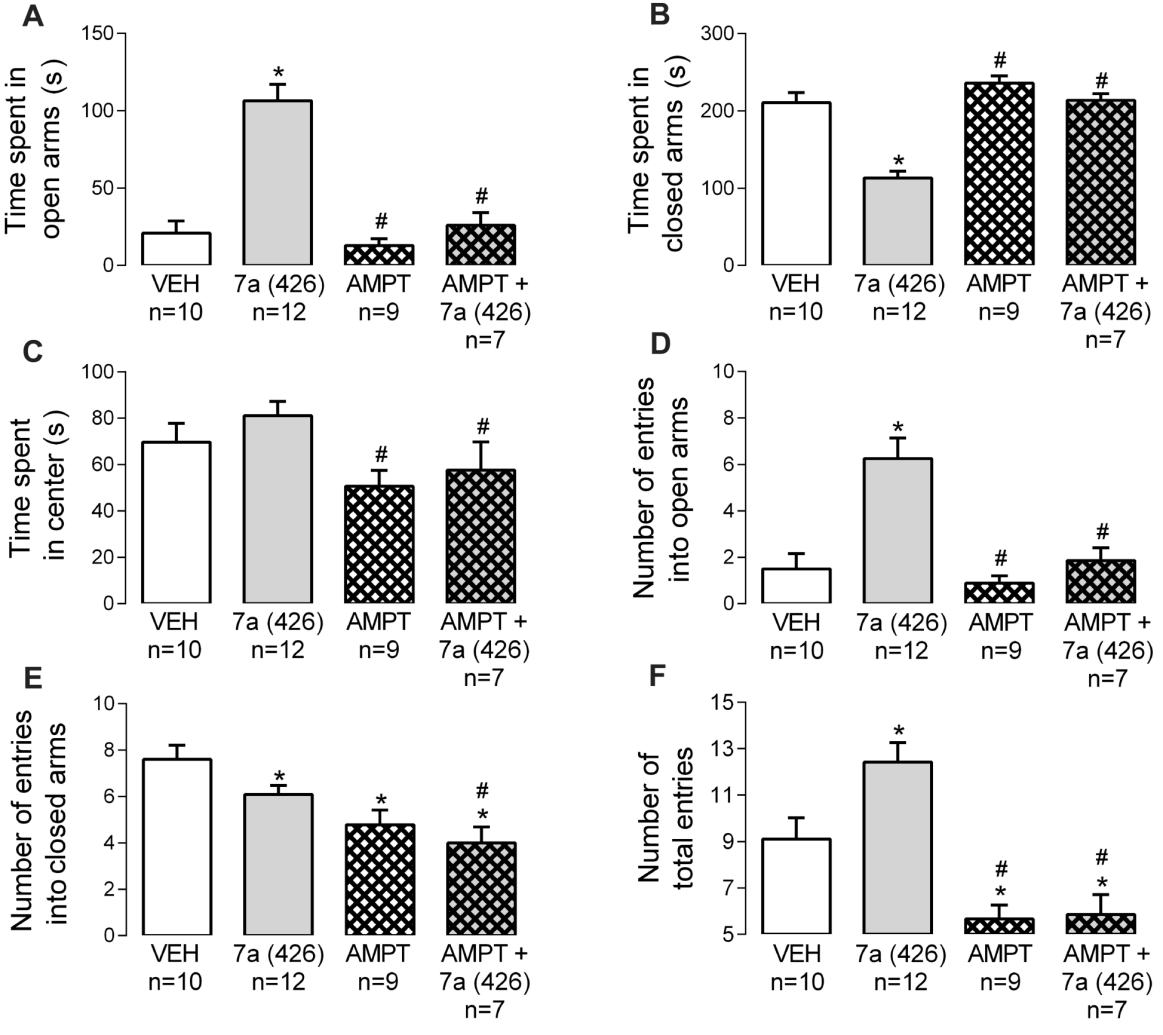
Contribution of catecholaminergic pathways to the anxiety-like effect evoked by Bj-PRO-7a in the elevated plus maze. **A**, Time spent in open arms; **B**, Time spent in closed arms; **C**, Time spent in center; **D**, Number of entries into open arms; **E**, Number of entries into closed arms; **F**, Number of total entries. Experimental groups: VEH: vehicle (0.9% NaCl); 7a (426): Bj-PRO-7a (426 nmol/kg); AMPT: α-methyl-DL-tyrosine (200 mg/kg); AMPT+7a (426): co-administration of α-methyl-DL-tyrosine and Bj-PRO-7a (426 nmol/kg). Data are reported as means±SE. *P<0.05 *vs* vehicle; ^#^P<0.05 *vs* 7a (426) (one-way analysis of variance followed by Fisher's *post hoc* test).

AMPT (200 mg/kg) reduced the locomotion in the EPM test compared to vehicle, as revealed by the decreases in number of entries into closed arms and number of total entries (Figure 5E and F). Additionally, catecholamine depletion promoted effects that were inverse to those evoked by *Bj*-PRO-7a, except in the number of entries into the closed arms of the maze (Figure 5F). Noticeably, the anxiolysis evoked by the heptapeptide was attenuated by simultaneous administration of α-methyl-DL-tyrosine with *Bj*-PRO-7a (Figure 5A, B, and D).

**Figure 6.**
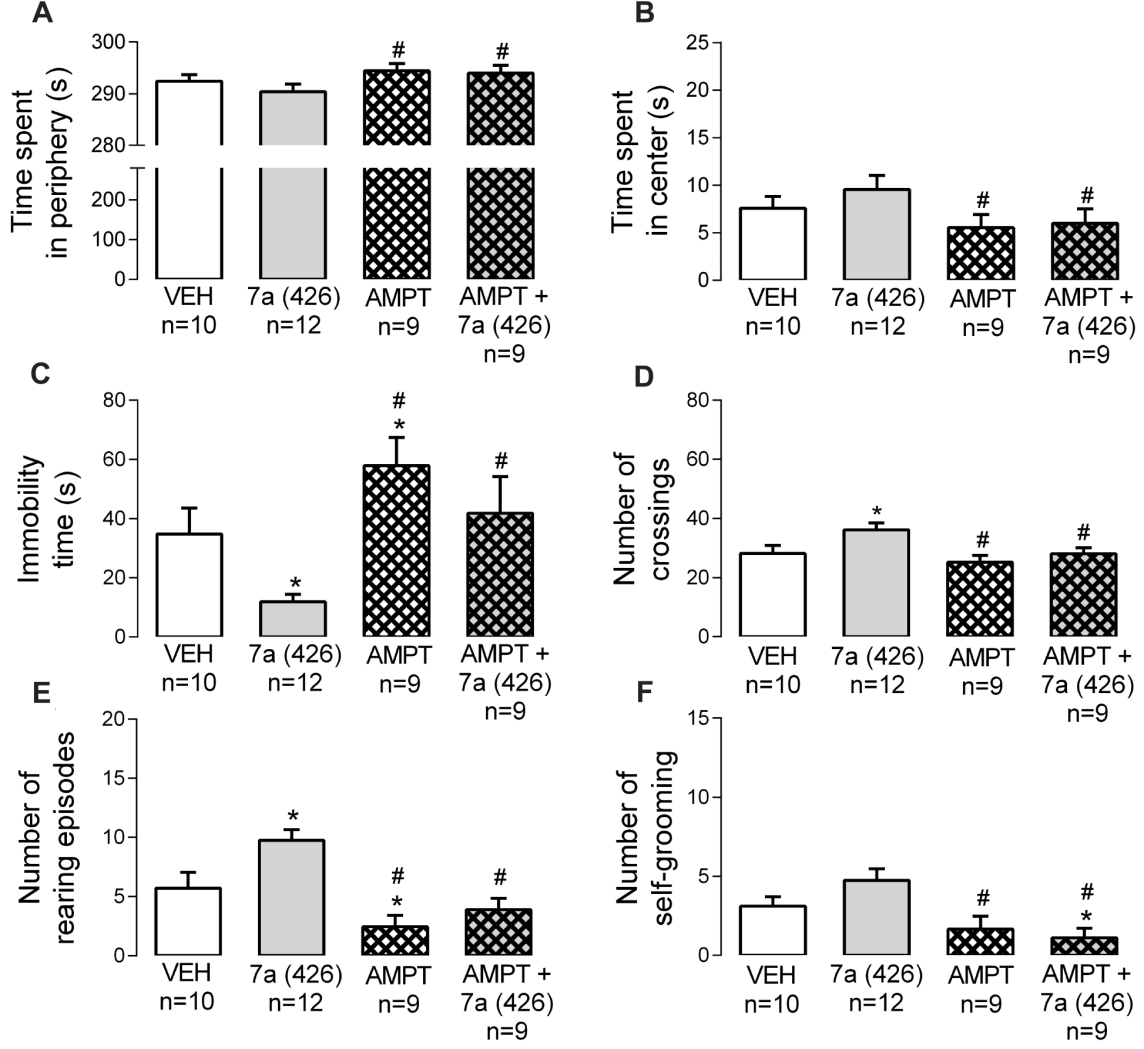
Contribution of catecholaminergic pathways (as revealed by the depleter α-methyl-DL-tyrosine) to the effects evoked by Bj-PRO-7a on locomotion/exploration in the open field. **A**, Time spent in periphery; **B**, Time spent in center; **C**, Immobility time; **D**, Number of crossings; **E**, Number of rearing episodes; **F**, Number of self-grooming espisodes. Experimental groups: VEH: vehicle (0.9% NaCl); 7a (426): Bj-PRO-7a (426 nmol/kg); AMPT: α-methyl-DL-tyrosine (200 mg/kg); AMPT + 7a (426): co-administration of α-methyl-DL-tyrosine and Bj-PRO-7a (426 nmol/kg). Data are reported as means±SE. *P<0.05 *vs* vehicle; ^#^P<0.05 *vs* 7a (426) (one-way analysis of variance followed by Fisher's *post hoc* test).

In OF, AMPT reduced the locomotion (Figure 6C) and exploration (Figure 6E) compared to vehicle. The catecholaminergic depletor promoted effects that were inverse to those evoked by the heptapeptide in locomotion and exploration parameters (Figure 6 C–F). Interestingly, the concomitant injection of AMPT and *Bj*-PRO-7a reverted the increases in locomotion and exploration evoked by the heptapeptide in the OF (Figure 6 C–F). These results suggested that catecholaminergic pathways are important for *Bj*-PRO-7a to perform their effects on locomotion and exploration.

### Dopaminergic receptors played a role in the behavioral effects evoked by *Bj*-PRO-7a

**Figure 7.**
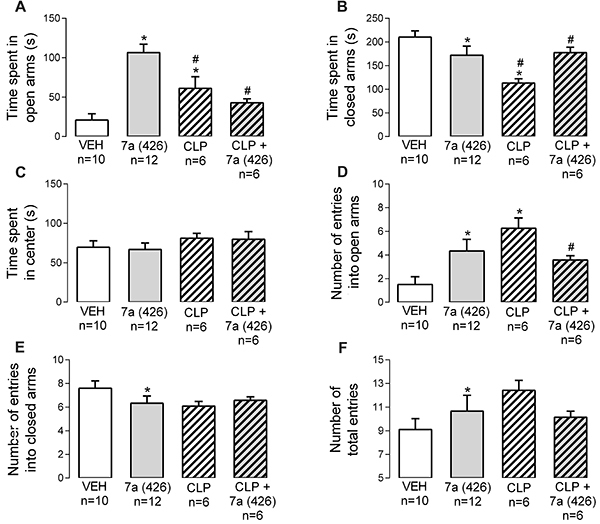
Contribution of dopaminergic receptors (as revealed by the antagonism with chlorpromazine) to the anxiety-like effect evoked by Bj-PRO-7a in the elevated plus maze. **A**, Time spent in open arms; **B**, Time spent in closed arms; **C**, Time spent in center; **D**, Number of entries into open arms; **E**, Number of entries into closed arms; **F**, Number of total entries. Experimental groups: VEH: vehicle (0.9% NaCl); 7a (426): Bj-PRO-7a (426 nmol/kg); CLP: chlorpromazine (2 mg/kg); CLP + 7a (426): co-administration of CLP and Bj-PRO-7a (426 nmol/kg). Data are reported as mean±SE. *P<0.05 *vs* vehicle; ^#^P<0.05 *vs* 7a (426) (one-way analysis of variance followed by Fisher's *post hoc* test).

CLP (2 mg/kg) promoted anxiolysis (Figure 7A, B, and D) compared to vehicle. This antagonism of dopaminergic receptors presented effects that were inverse to those evoked by *Bj*-PRO-7a in time spent in open and closed arms of the maze (Figure 7A and B). Intriguingly, the anxiolysis evoked by *Bj*-PRO-7a was reverted by its concomitant administration with CLP, as revealed by decreases in the time spent in the open arms and the number of entries into the maze area (Figure 7A and D). Therefore, the activity of dopaminergic receptors was required to promote the anxiolysis evoked *Bj*-PRO-7a.

**Figure 8.**
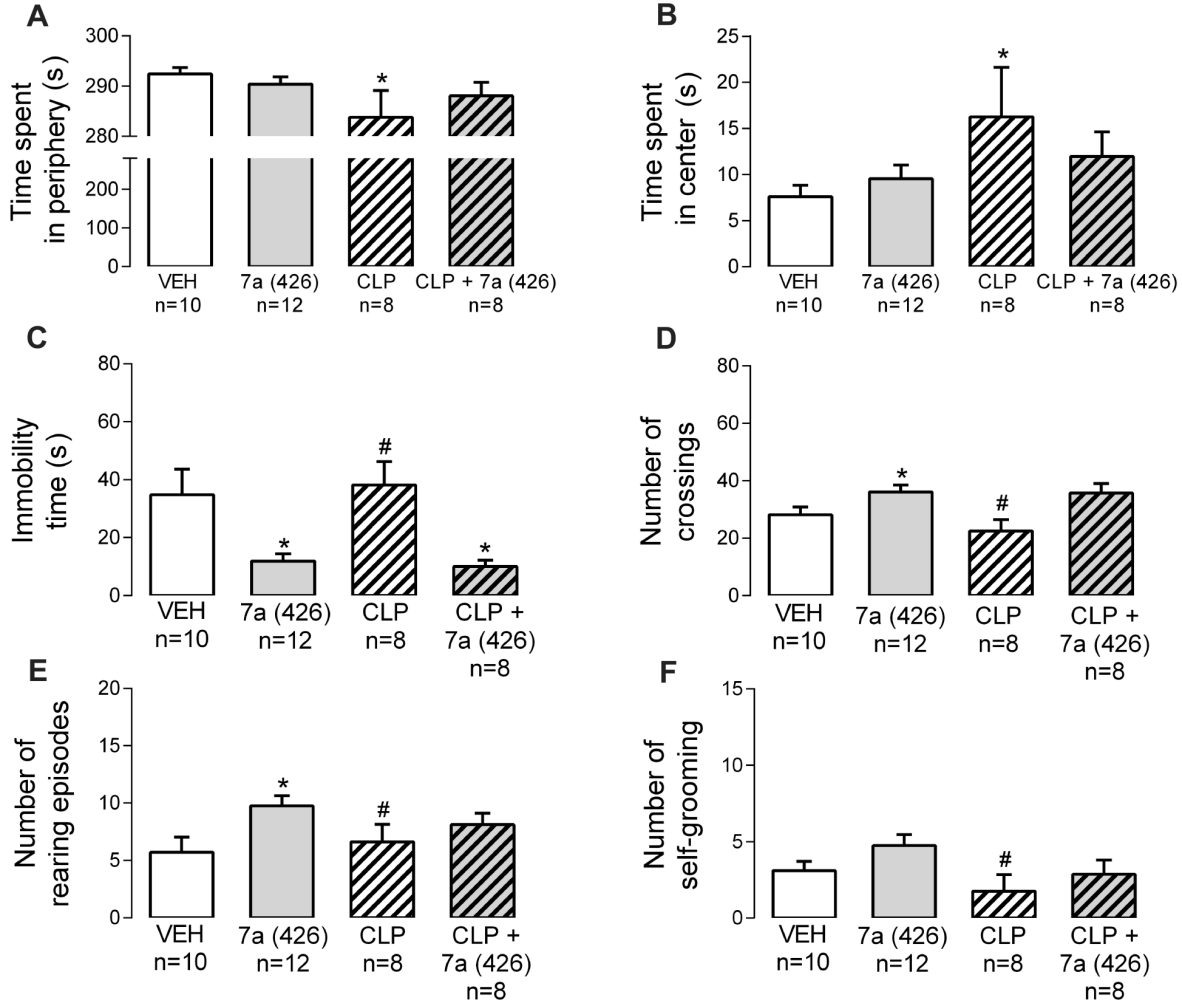
Contribution of dopaminergic receptors (as revealed by the antagonism with chlorpromazine) to the effects evoked by Bj-PRO-7a on locomotion/exploration in the open field. **A**, Time spent in periphery; **B**, Time spent in center; **C**, Immobility time; **D**, Number of crossings; **E**, Number of rearing episodes; **F**, Number of self-grooming episodes. Experimental groups: VEH: vehicle (0.9% NaCl); 7a (426): Bj-PRO-7a (426 nmol/kg); CLP: chlorpromazine (2 mg/kg); CLP + 7a (426): co-administration of CLP and Bj-PRO-7a (426 nmol/kg). Data are reported as means±SE. *P<0.05 *vs* vehicle; ^#^P<0.05 *vs* 7a (426) (one-way analysis of variance followed by Fisher's *post hoc* test).

In the OF, CLP provoked anxiolytic-like effects compared to vehicle (Figure 8A and B). Also, the antagonism of dopaminergic receptors reduced the locomotion and exploration activities compared to *Bj*-PRO-7a (Figure 8C–F). Intriguingly, the co-administration of CLP with *Bj*-PRO-7a reduced the immobility compared to vehicle. Conversely to what was found in the EPM, dopaminergic receptors did not play a role in the locomotion and exploratory effects evoked by *Bj*-PRO-7a (Figure 8C-F).

### Antidepressant-like actions of *Bj*-PRO-7a depended on catecholaminergic synthesis and on activation of M_1_R

**Figure 9.**
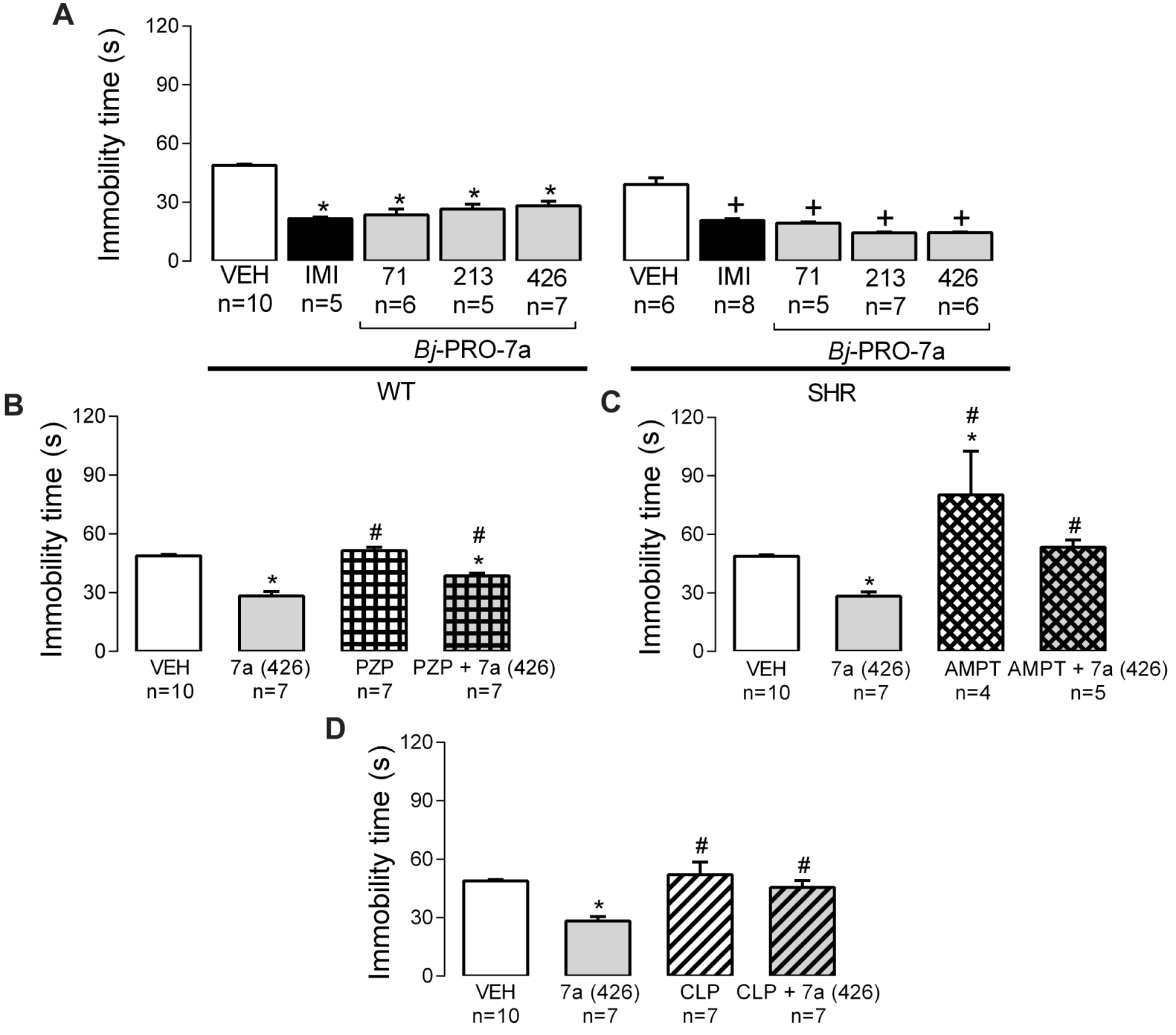
Antidepressant-like effects measured by the immobility time in the forced swimming test. **A**, Effects of Bj-PRO-7a on antidepressant-like behaviors in Wistar (WT) and spontaneously hypertensive rats (SHR). **B**, Contribution of muscarinic receptor subtype 1 (as revealed by the antagonism with pirenzepine - PZP) to the anti-depressant-like effects evoked Bj-PRO-7a. **C**, Contribution of catecholaminergic paths (as revealed by the depleter α-methyl-DL-tyrosine: AMPT) to the effects evoked by Bj-PRO-7a. **D**, Contribution of dopaminergic receptors (as revealed by the antagonism with chlorpromazine - CLP) to the effects evoked by Bj-PRO-7a. Experimental groups: VEH: vehicle (0.9% NaCl); IMI: imipramine (15 mg/kg); 7a: Bj-PRO-7a (71, 213, or 426 nmol/kg); PZP: pirenzepine (852 nmol/kg); PZP + 7a(426): co-administration of PZP and Bj-PRO-7a (426 nmol/kg); AMPT (200 mg/kg); AMPT+7a(426): co-administration of AMPT and Bj-PRO-7a (426 nmol/kg); CLP: CLP (2 mg/kg); CLP+7a (426): co-administration of CLP and Bj-PRO-7a (426 nmol/kg). Data are reported as means±SE. *P<0.05 *vs* VEH WT; ^+^P<0.05 *vs* VEH SHR; ^#^P<0.05 *vs* 7a (426) [two-way analysis of variance followed by Bonferroni's test (panel **A**) and one-way analysis of variance followed by Fisher's *post hoc* test (panels **B**-**D**)].

As expected, the treatment with imipramine – a tricyclic antidepressant – reduced the immobility time compared to vehicle (Figure 9A). The treatment with *Bj*-PRO-7a at doses of 71, 213, and 426 nmol/kg reduced the immobility time (Figure 9A) in normotensive and hypertensive rats during the FS test compared to vehicle. Considering that *Bj*-PRO-7a produced the same antidepressant-like effect in both strains (normotensive and antihypertensive rats), the subsequent trials were conducted only in WT rats with vehicle and *Bj*-PRO-7a (426 nmol/kg) used to compare the effects of PZP, AMPT, and CLP.

The immobility time sampled following M_1_R antagonism with PZP did not differ from vehicle (Figure 9B). Interestingly, the co-administration of PZP with *Bj*-PRO-7a decreased the immobility time compared to vehicle (Figure 9B), but these values were still greater than those produced by *Bj*-PRO-7a. Therefore, the antidepressant-like effects promoted by the heptapeptide partially depend on M_1_R.

Catecholaminergic depletion with AMPT increased the immobility time in the FS test (Figure 9C). Consequently, this catecholaminergic depletion was able to revert the antidepressant-like effect evoked by *Bj*-PRO-7a.

The antagonism of dopaminergic receptors with CLP did not modify the magnitude of the immobility time measured in the group injected vehicle (Figure 9D). However, compared to *Bj*-PRO-7a, CLP increased the immobility time. In this regard, the co-administration of CLP and *Bj*-PRO-7a decreased the amplitude of the antidepressant-like effect evoked by the heptapeptide, since the results obtained from this co-administration were comparable to vehicle (Figure 9D). Therefore, the antidepressant-like effects evoked by *Bj*-PRO-7a relied on dopaminergic receptors.

## Discussion

The main findings of this study were: i) *Bj*-PRO-7a promoted anxiolytic and antidepressant-like effects in WT and SHRs; ii) the higher dose of the heptapeptide increased the locomotion and exploration of rats; iii) the antagonism of M_1_R by PZP reverted the anxiolytic-like effect and decreased the antidepressant-like effect evoked by *Bj*-PRO-7a; iv) catecholamines depletion, a result of inhibition of tyrosine hydroxylase activity by AMPT, reverted the anxiolytic-like and antidepressant-like effects caused by *Bj*-PRO-7a; and v) the antagonism of dopaminergic receptors by CLP reverted the anxiolytic-like effect and attenuated the antidepressant-like effect provoked by *Bj*-PRO-7a.

Snake venoms are cocktails of toxins composed by enzymatic and non-enzymatic bioactive polypeptides and in less proportion, carbohydrates, lipids, amino acids, and minerals. The side effects triggered from envenoming by *Bothrops jararaca* bite are severe pain, acute renal failure, shock, intracranial hemorrhage, and other hemostatic disorders. The toxins displaying enzymatic activities, such as hydrolases, phosphodiesterases, and phospholipases, are known for converging actions towards prey immobilization and the subsequent intake and digestion. These enzymatic toxins may be either promiscuous or highly specific with regard to their molecular targets (2). However, the effects of these neuroactive compounds are not confined to the somatic nervous system, thus affecting behavior and other functions by crossing the blood-brain barrier and, as a consequence, these toxins are able to reach the central nervous system (CNS) (2). Furthermore, neuroactive compounds have a potent curarimimetic action on peripheral cholinergic transmission (14). Considering the multiplicity of venom components and the molecular targets, it is plausible to hypothesize that venoms previously known for being predominantly composed by hemotoxins would also be able to reach a degree of neurotoxicity.

In the case of the peptides from *Bj* venom, studies demonstrated that a peptide that is structurally similar to *Bj*-PRO-7a, the *Bj*-PRO-10c, was detected in mice brain following its peripheral injection. They observed that *Bj*-PRO-10c was found at 5 and 60 min following its intraperitoneal injection (7). The route of injection and the timeframe of our experiments were similar to those employed in that study. About 2% of *Bj*-PRO-10c was found in brain, which allowed drawing some hypotheses that should be considered: i) this 2% would be enough to produce the effects we found; ii) *Bj*-PRO-7a is smaller compared to *Bj*-PRO-10c, and it may increase the possibilities of crossing the blood-brain barrier; iii) the peptide is able to cross the blood-brain barrier because of four benzene rings in its primary structure, which is strongly correlated with a degree of liposolubility (see Supplementary Figure S1); iv) M_1_R are broadly distributed in brain areas well known for controlling behavior, such as prefrontal cortex, amygdala, hippocampus, and striatum (15,16), and the reversion of the *Bj*-PRO-7a-evoked anxiolysis by PZP is further evidence of a central effect for the heptapeptide. Therefore, we believe that *Bj*-PRO-7a can change brain function and these changes partially depend on central M_1_R.

Seminal studies already proposed the cholinergic system as one of the targets for snake toxins (14). Cholinergic neurons are broadly distributed in the CNS and modulate behavior and the cardiovascular and neuroendocrine systems (17). The central cholinergic system has been implicated in the physiopathology of affective disorders, mood regulation, sleep, and autonomic responses to stress (18). Besides, the central cholinergic system is able to affect – directly or indirectly – the function of other systems, such as the catecholaminergic. Dilsaver and Greden (1984) described that the activity of the tyrosine hydroxylases, the enzymatic limiting step for neuronal production of dopamine, adrenaline, and noradrenaline, is modulated by cholinergic mechanisms (19). In fact, the stimulation of cholinergic muscarinic receptors (mAChRs) reduces noradrenaline (NE) release in the hypothalamus (20), a highly permeable diencephalic region that may be considered a behavioral-autonomic interface. Additionally, mAChRs activation induces production and release of neurotrophic factors in neurons (21), which are important to reach antidepressant effects by producing synaptic plasticity, axon outgrowth, and neurogenesis. Therefore, the aforementioned reports are robust enough to justify the search for molecules that modulate cholinergic and catecholaminergic systems, since these paths are extremely important to the neurobiological organization of different functions and behaviors.

The function of central mAChRs varies according with the neural substrate in which they are expressed and with the subtype of these receptors. The mAChRs subtypes 1, 3, and 5 are attached to Gq protein with excitatory function. Conversely, the mAChRs subtypes 2 and 4 are attached to Gi protein, with inhibitory function (22). Gerber and colleagues showed the functional importance of M_1_R to the dopaminergic neurotransmission and to the control of locomotion. They found a positive correlation between the increases in M_1_R expression and release of dopamine in striatum (23). Much of this evidence was obtained using behavioral paradigms, which supports the M_1_R involvement in anxiety, depression, and in other neuronal disorders affecting behavior. A previous *in vitro* study (8) associated the cardiovascular effects of *Bj*-PRO-7a to M_1_R. In light of these mechanistic clues, we tested the hypothesis that the anxiolytic and antidepressant-like effects of *Bj*-PRO-7a would rely on M_1_R. We found that behavioral effects evoked by *Bj*-PRO-7a were either reverted or attenuated by the simultaneous administration with PZP, which confirmed that the *Bj*-PRO-7a activity on behavior relies on M_1_R. However, the reductions in the immobility time evoked by *Bj*-PRO-7a in the OF were unaffected by the concomitant antagonism of M_1_R with PZP, which demonstrates the involvement of other pathways and synapses in the effects of *Bj*-PRO-7a on locomotion/exploration. Therefore, the subsequent question to be answered was about the other targets through which *Bj*-PRO-7a produces behavioral effects.

In order to uncover whether catecholaminergic pathways would be involved in the effects evoked by *Bj*-PRO-7a, the methodological choice was to inhibit the tyrosine hydroxylase enzyme by AMPT. As a consequence, substantial depletions in dopamine, noradrenaline, and adrenaline levels, – as the natural sequence of reactions in the catecholaminergic metabolic paths – are found following injection of this enzymatic inhibitor (24). Our results showed that catecholaminergic pathways were substantially involved in the *Bj*-PRO-7a effects on anxiety and depression-like behaviors. The overactivation of the noradrenergic system can promote anxiety, especially due to malfunctioning of limbic structures (25). The *locus coeruleus* is the major brain source of noradrenergic neurons in charge of responding to stressors. Cholinergic neurons of the reticular system and gigantocellular tegmental area interact with *locus coeruleus*, modulating NE release (17). The projections from amygdala to *locus coeruleus* (26) is another possible pathway underlying interaction between central cholinergic and noradrenergic systems. Additionally, M_1_Rs are expressed in basolateral amygdala, an important area involved in the sensorial circuitries (27). Therefore, the cholinergic system can regulate the activity of the noradrenergic system and consequently the organization of defense reactions. Molecules that are capable of attenuating increases in the firing rate of noradrenergic neurons in *locus coeruleus* are effective in reducing anxiety levels (28). In regard to our results, it is worth considering that the heptapeptide may regulate the NE release with consequent decreases in responsiveness to stress, thus allowing the rat to explore aversive areas. Notwithstanding the possible involvement of M_1_Rs in anxiety, our results indicated that the activity of these receptors did not appear to contribute directly to the attenuation of depressive behavior exerted by *Bj*-PRO-7a.

Since dopamine is the major player in motivational processes that are impaired in mental disorders (29), we decided to investigate the contribution of dopaminergic receptors to the effects evoked by the heptapeptide. Despite finding that the antagonism of dopaminergic receptors with CLP itself promoted anxiolytic-like effect, the anxiolytic and antidepressant-like effects of *Bj*-PRO-7a were reverted by the concomitant injection with CLP. The hypotheses that can be built from these observations are: i) the functionality of M_1_R implicates directly in dopamine release (23); ii) dopaminergic activity contributes to attenuate anxiety – probably by dopaminergic receptor subtype 2 – and depression; iii) *Bj*-PRO-7a would increase dopamine transmission on mesolimbic and mesocortical systems related to anxiolysis, motivation, exploration, and antidepressant effects (30).

Systems other than the noradrenergic, dopaminergic, and cholinergic may be involved in *Bj*-PRO-7a effects on behavior. Glutamatergic, gabaergic, and oxidonitrergic pathways would be good candidates for further studies. These hypotheses become even more plausible when previous reports on *Bj*-PRO-7a effects are kept in mind: i) the modulation of calcium transients in neurons (31) and ii) the activation of oxidonitrergic intracellular pathways by M_1_R agonism (8). Central NO increases extracellular concentrations of glutamate and γ-aminobutyric acid (GABA) and regulates ACh and serotonin release in some brain regions governing locomotion and cognition (32). Interestingly, several antidepressants are capable of modulating central oxidonitrergic activity, such as serotonin reuptake inhibitors (33). The possibility that oxidonitrergic paths are recruited by *Bj*-PRO-7a to reach the antidepressant and anxiolytic-like effects is supported by previous findings: *Bj*-PROs are able to activate NO release (4). Therefore, it cannot be neglected that central NO and GABA may be involved in anxiolysis and antidepressant-like effects of *Bj*-PRO-7a. This, however, needs to be addressed in further studies.

Our study indicated that the activation of cholinergic muscarinic receptors subtype 1 is required to reach the anxiolytic-like effect evoked by *Bj*-PRO-7a. The anxiolytic- and antidepressant-like effects of *Bj*-PRO-7a seem to depend on catecholaminergic pathways and on dopamine receptors. Interestingly, the M_1_Rs activation contributes to enhance memory and cognitive processes by inducing long-term potentials in hippocampus (34). Therefore, *Bj*-PRO-7a appears to be a promising innovative prototype capable of simultaneously treating the most prevalent psychiatric diseases such as anxiety and depression, through alternative mechanistic pathways with less adverse effects, contrary to many commercially available drugs often known for causing side effects such as drowsiness, hypnotic effects, cognitive and motor deficits, mental confusion, tolerance, and dependence (35). In spite of the noticeable *Bj*-PRO-7a cardiovascular effects, it is important to emphasize that the heptapeptide was able to reduce the blood pressure of hypertensive rats, i.e., the heptapeptide does not promote hypotension in the normotensive strain (6). Altogether, these central and peripheral effects indicate *Bj*-PRO-7a to be a good prototype candidate for treating disorders involving cholinergic and catecholaminergic targets.

## Supplementary Material

Click here to view [pdf].

## References

[1] WHO (World Health Organization) (2018). Mental Health Atlas..

[2] Osipov AV, Utkin YN, Gopalakrishnakone P, Inagaki H, Vogel CW, Mukherjee A, Rahmy T, editors. (2017). Snake venom toxins targeted at the nervous system.

[3] Camargo AC, Ianzer D, Guerreiro JR, Serrano SM (2012). Bradykinin-potentiating peptides: beyond captopril. Toxicon.

[4] Morais KLP, Ianzer D, Miranda JRR, Melo RL, Guerreiro JR, Santos RA (2013). Proline rich-oligopeptides: Diverse mechanisms for antihypertensive action.. Peptides.

[5] Ianzer D, Konno K, Marques-Porto R, Vieira Portaro FC, Stocklin R, Camargo AC (2004). Identification of five new bradykinin potentiating peptides (BPPs) from Bothrops jararaca crude venom by using electrospray ionization tandem mass spectrometry after a two-step liquid chromatography. Peptides.

[6] Ianzer D, Santos RA, Etelvino GM, Xavier CH, de Almeida Santos J, Mendes EP (2007). Do the cardiovascular effects of angiotensin-converting enzyme (ACE) I involve ACE-independent mechanisms? New insights from proline-rich peptides of Bothrops jararaca. J Pharmacol Exp Ther.

[7] Silva CA, Portaro FC, Fernandes BL, Ianzer DA, Guerreiro JR, Gomes CL (2008). Tissue distribution in mice of BPP 10c, a potent prolinerich anti-hypertensive peptide of Bothrops jararaca. Toxicon.

[8] Negraes PD, Lameu C, Hayashi MA, Melo LM, Camargo AC, Ulrich H (2011). The snake venom peptide Bj-Pro-7a Is a M1 muscarinic acetylcholine receptor agonist. Cytometry A.

[9] Morilak DA, Frazer A (2004). Antidepressants and brain monoaminergic systems: a dimensional approach to understanding their behavioural effects in depression and anxiety disorders. Int J Neuropsychopharmacol.

[10] da Cruz KR, Turones LC, Camargo-Silva G, Gomes KP, Mendonça MM, Galdino P (2017). The hemoglobin derived peptide LVV-hemorphin-7 evokes behavioral effects mediated by oxytocin receptors. Neuropeptides.

[11] Witkin JM, Overshiner C, Li X, Catlow JT, Wishart GN, Schober DA (2014). M1 and M2 muscarinic receptor subtypes regulate antidepressant-like effects of the rapidly acting antidepressant scopolamine. J Pharmacol Exp Ther.

[12] Degroot A, Nomikos GG (2005). Fluoxetine disrupts the integration of anxiety and aversive memories. Neuropsychopharmacol.

[13] Terzioglu B, Kaleli M, Aydın B, Ketenci S, Cabadak H, Goren MZ (2013). Increased noradrenaline levels in the rostral pons can be reversed by M1 Antagonist in a rat model of post-traumatic stress disorder. Neurochem Res.

[14] Chang CC, Lee CY (1963). Isolation of neurotoxins from the venom of bungarus multicinctus and their modes of neuromuscular blocking action. Arch Int Pharmacodyn Ther.

[15] Flynn DD, Ferrari-DiLeo G, Mash DC, Levey AI (1995). Differential regulation of molecular subtypes of muscarinic receptors in Alzheimer's disease. J Neurochem.

[16] Levey AI, Edmunds SM, Koliatsos V, Wiley RG, Heilman CJ (1995). Expression of m1-m4 muscarinic acetylcholine receptor proteins in rat hippocampus and regulation by cholinergic innervation. J Neuroscience.

[17] Dilsaver SC (1986). Cholinergic mechanisms in affective disorders. Acta Psychiatr Scand.

[18] Dilsaver SC (1986). Cholinergic mechanisms in depression. Brain Res.

[19] Dilsaver SC, Greden JF (1984). Antidepressant withdrawalinduced activation (hypomania and mania): mechanism and theoretical significance. Brain Res.

[20] Westfall TC, Usdin E, Snyder SH (1973). Effect of acetylcholine on the release of (^3^H)norepinephrine by nicotine and potassium chloride from rat brain slices. Frontiers of catecholamine research.

[21] Navakkode S, Korte M (2012). Cooperation between cholinergic and glutamatergic receptors are essential to induce BDNF-dependent long-lasting memory storage. Hippocampus.

[22] Eglen RM (2012). Overview of Muscarinic Receptor Subtypes. Handb Exp Pharmacol.

[23] Gerber DJ, Sotnikova TD, Gainetdinov RR, Huang SY, Caron MG, Tonegawa S (2001). Hyperactivity, elevated dopaminergic transmission and response to amphetamine in M1 muscarinic acetylcholine receptor-deficient mice. Proc Natl Acad Sci USA.

[24] Corrodi H, Hanson LC (1966). Central effects of an inhibitor of tyrosine hydroxylation. Psychopharmacologia.

[25] Lange C, Irle E (2004). Enlarged amygdala volume and reduced hippocampal volume in young women with major depression. Psychol Med.

[26] Jacobs BL, Abercrombie ED, Fornal CA, Levine ES, Morilak DA, Stafford IL (1991). Single-unit and physiological analyses of brain norepinephrine function in behaving animals. Prog Brain Res.

[27] LeDoux JE (2000). Emotion circuits in the brain. Annu Rev Neurosci.

[28] Bremner JD, Krystal JH, Southwick SM, Charney DS (1996). Noradrenergic mechanisms in stress and anxiety: I. Preclinical studies. Synapse.

[29] Pignatelli M, Bonci A (2015). Role of dopamine neurons in reward and aversion: a synaptic plasticity perspective. Neuron.

[30] Rezayof A, Hosseini SS, Zarrindast MR (2009). Effects of morphine on rat behaviour in the elevated plus maze: the role of central amygdala dopamine receptors. Behav Brain Res.

[31] Lameu C, Hayashi MA, Guerreiro JR, Oliveira EF, Lebrun I, Pontieri V (2010). The central nervous system as target for antihypertensive actions of a proline-rich peptide from Bothrops jararaca venom. Cytometry A.

[32] Segovia G, Porras A, Mora F (1994). Effects of a nitric oxide donor on glutamate and GABA release in striatum and hippocampus of the conscious rat. Neuroreport.

[33] Dhir A, Kulkarni SK (2011). Nitric oxide and major depression. Nitric Oxide.

[34] Dennis SH, Pasqui F, Colvin EM, Sanger H, Mogg AJ, Felder CC (2016). Activation of muscarinic M1 acetylcholine receptors induces long-term potentiation in the hippocampus. Cereb Cortex.

[35] Ashton AK, Jamerson BD, L Weinstein W, Wagoner C (2005). Antidepressant-related adverse effects impacting treatment compliance: results of a patient survey. Curr Ther Res Clin Exp.

